# From the laboratory bench to the lecture hall: latest findings from mycotoxin research presented at the 43rd Mycotoxin Workshop

**DOI:** 10.1007/s12550-022-00462-2

**Published:** 2022-07-15

**Authors:** Olivier Puel, Benedikt Cramer, Isabelle P. Oswald, Hans-Ulrich Humpf

**Affiliations:** 1 Toxalim (Research Centre in Food Toxicology), Université de Toulouse, INRAE, ENVT, INP-Purpan, UPS, Toulouse, France; 2 Institute of Food Chemistry, Westfälische Wilhelms-Universität, Corrensstrasse 45, 48149 Münster, Germany

After 2 years of COVID restrictions, with no Mycotoxin Workshop in 2020 and an online only workshop last year, around 180 scientists from 21 countries met in person at the 43rd Mycotoxin Workshop in Toulouse, France, from May 30 to June 1, 2022. The workshop was organized on behalf of the *Society for Mycotoxin Research* by several research groups from Toulouse led by Dr. Isabelle P. Oswald and Dr. Olivier Puel and their team “Biosynthesis and Toxicity of Mycotoxins” at the Research Center in Food Toxicology, TOXALIM, INRAE, in cooperation with the Chemical Engineering Laboratory, the Purpan Engineering School and the Pharma-DEV laboratory. The 43rd Mycotoxin Workshop took place at the Hôtel-Dieu Saint-Jacques, which is an ancient hospital from the twelfth century beautifully situated on the left bank of the Garonne River.

During the opening ceremony, the president of the *Society for Mycotoxin Research*, Prof. Dr. Hans-Ulrich Humpf, welcomed all participants and emphasized that the *Society* is a non-profit organization focusing on scientific exchange in the field of mycotoxin research and supporting particularly young scientist. Members benefit from a free print subscription of the official journal of the *Society*, *Mycotoxin Research*, and are entitled to a discounted registration fee for the Mycotoxin Workshop. The head of the Research Center in Food Toxicology, Isabelle P. Oswald, also warmly welcomed all participants with some information about Toulouse, details about the scientific and social program, and great thanks to the local organizing team as well as all sponsors supporting the Workshop.

During the scientific program, especially, young scientists presented their latest mycotoxin research results directly from the lab bench in 37 lectures and 83 poster presentations. The following research topics were covered in 8 sessions: (1) occurrence and analyses of mycotoxins in food, feed, and physiological samples; (2) genetics, biology, and new secondary metabolites of toxigenic fungi; (3) effects of mycotoxins on human and animal health; (4) toxicity and risk assessment; and (5) food and feed safety. The scientific program covered new challenging topics, such as the contamination of new feed material, the health aspects of mycotoxins on salmon due to grain-based feed, human biomonitoring, and co-exposure scenarios of mycotoxins with other contaminants such as acrylamide. Other presentations addressed metabolomics approaches to assess toxic effects of mycotoxins, insights into JECFA risk assessment, and mimicking the influence of climate change scenarios on aflatoxin production. In a frame of the scientific tradition, the analytical methods for the determination of mycotoxins in various matrices were a key topic. It was demonstrated that approaches with sensitive mass spectrometry techniques might answer complex questions, such as the mode of action of mycotoxins and their precursors in various cell culture models. Along with in vitro techniques, several in silico methods were presented and discussed. Computational tools have much evolved in recent years and could be successfully used not only for toxicokinetic studies but also for genotoxicity investigations. Besides the major — and well known — negative aspects of mycotoxins on human and animal health, new research fields aiming at the use of mycotoxins (and derivatives thereof) as new, future therapeutic agents were also presented.

The scientific programme was accompanied by several, really great social events. These have a long tradition in Mycotoxin Workshops, in accordance with the aim of the *Society* to provide a platform for deepening scientific discussions, creating new contacts between researchers from different countries, and initiating new scientific collaborations. Besides the excellent French finger food for lunch, a walking tour in the city of Toulouse, a boat trip on the Canal du Midi, and an outstanding gala dinner at Hôtel-Dieu Saint-Jacques were provided by the local organizers. During the closing session, all participants thanked the organizers with standing ovations and long applause for the excellent conference and some splendid days in Toulouse.

## General meeting of the *Society for Mycotoxin Research*

During the annual meeting of the *Society for Mycotoxin Research*, the president, Hans-Ulrich Humpf, gave a report on the recent activities of the *Society*, including the proceedings of the meetings of the board of directors during 2021 and 2022. The scientific highlight last year was the 42nd Mycotoxin Workshop, first ever online conference of the *Society*, organized from the Institute of Food Chemistry at the University of Muenster, with around 300 participants from 31 countries (Kalinina et al. 2021).

During the regular meetings of the board of the *Society*, several challenges had to be solved. First, the Editor in Chief (EiC) of the *Society’s* own scientific journal *Mycotoxin Research*, Prof. Dr. Ewald Usleber, informed the board that he would like to step back from his position after holding it for 15 years. Finding a new EiC was a demanding task, but after intensive internal and external discussions, Hans-Ulrich Humpf agreed to take over this position from January 1, 2023. At the same time, he then will step back from his current position as president of the *Society*. According to the bylaws of the *Society*, the board decided that the current vice-president, Prof. Dr. Madeleine Plötz (University of Veterinary Medicine Hannover, Germany), should take over the position of the president of the *Society*. The board of the *Society* is happy to have Dr. hab. Magdalena Twarużek, university professor (Kazimierz Wielki University, Poland), as a new vice-president, until the next official election in 2023, and hopefully beyond that (according to the bylaws, the board of directors is elected for 2 years). As this is a major change of the board, the members of the *Society* were asked for their vote, and they unanimously supported the suggested composition of the new board.

Furthermore, the president reported that the former president of the *Society*, Prof. Dr. habil. Manfred Gareis was elected as an honorary member of the *Society* by the board for his long-lasting activity in the board, acknowledging his outstanding support of the *Society*.

As the homepage of the *Society* did not fulfill current standards, for example with regard to safety, a new homepage was brought online with the great support of Dr. Carsten Krischek. The new internet portal of the *Society* can be found at https://www.mycotoxin.de/ and for the Mycotoxin Workshops: https://www.mycotoxin-workshop.eu/. The websites are still under construction and will be continuously improved.

The treasurer of the *Society*, Dr. Benedikt Cramer, gave a short report about the financial situation, which overall developed positively, thanks to reductions in personal costs and a significant gain at the last Workshop.

Ewald Usleber, the current EiC of *Mycotoxin Research*, gave a short report about the current situation of the journal. *Mycotoxin Research* and its impact on the scientific community continue to develop very well. In its 38th year of continuous publication, the *Society* can proudly claim that *Mycotoxin Research* enjoys an increasingly well reception within the scientific community. For example, the current impact factor (Clarivate Analytics) for 2021 is now 4.082. *Mycotoxin Research* is covered by all major scientific databases worldwide, and online access is guaranteed by our publisher, Springer/Nature. Ewald closed his final report as the EiC with thanking the authors, editorial board members, reviewers, and the publishers of *Mycotoxin Research*. Without their perseverance, and without the continued support from the membership of the *Society*, the success story of *Mycotoxin Research* would not have been possible.

## Announcement: 44th Mycotoxin-Workshop 2023 in Hannover, Germany

The next and 44th Mycotoxin-Workshop will take place on June 5–7, 2023, in Hannover, Germany, and will be organized by the group of Madeleine Plötz from the University of Veterinary Medicine Hannover in cooperation with the *Society for Mycotoxin Research*. Further information will be available at the website of the *Society for Mycotoxin Research* (www.mycotoxin.de).
